# Objective and perceived availability of physical activity opportunities: differences in associations with physical activity behavior among urban adolescents

**DOI:** 10.1186/1479-5868-6-70

**Published:** 2009-10-15

**Authors:** Richard G Prins, Anke Oenema, Klazine van der Horst, Johannes Brug

**Affiliations:** 1 Department of Public Health, Erasmus University Medical Center, Rotterdam, the Netherlands; 2 ETH Zürich, Institute for Environmental Decisions (IED), Consumer Behavior, Zürich, Switzerland; 3 EMGO Institute for Health and Care Research, VU University Medical Center, Amsterdam, the Netherlands

## Abstract

**Background:**

This study examined the associations of the perceived and objective environment with adolescent engagement in sports activities and walking and cycling in leisure time. It also explored the degree of agreement between objective and perceived availability of physical activity (PA) facilities in neighborhoods.

**Methods:**

Cross-sectional data on physical activity, the perceived availability of physical activity opportunities (perceived physical environment) was assessed through a questionnaire and the objective availability of PA opportunities (objective physical environment) was obtained through GIS data. The final sample included 654 adolescents with a mean age of 14.1 (SD = 1.2) years.

**Results:**

Perceived availability of sports facilities and parks was significantly associated with engaging in sports (OR: 1.73; 95% CI: 1.16-2.56) and with walking and cycling in leisure time (OR: 1.66; 95% CI: 1.07-2.57) respectively. Agreement between objective and perceived environment was low to moderate with Kappa values ranging from -0.005 to 0.053.

**Conclusion:**

The perceived environment was the stronger correlate of PA behavior among adolescents. There were substantial differences between assessments of objective and perceived physical environment.

## Background

Insufficient physical activity (PA) is one of the major risk factors for chronic diseases such as cardiovascular diseases, cancer and obesity [1,2]. The Dutch PA guidelines state that adolescents have to engage in moderate-intensity PA for at least one hour each day [3,4], and to engage at least three times a week for at least 20 minutes in vigorous intensity activities such as sports ("fitness norm") [5]. Only 27% of Dutch adolescents meet this guideline [6], and 34% meet the "fitness norm". Similar figures have been found in other Western countries [5,7-10]. Increasing PA is therefore important for population health. Adolescents are a particularly important group to target, since sufficient PA can result in considerable health gains for this group. The health benefits of regular PA for adolescents include a lower risk of becoming overweight or obese [2,11], higher bone density [2,11], a lower risk of depression [2] and healthier cardiovascular risk profiles [11]. These benefits may be experienced earlier as well as later in life [12]. Furthermore, physically active adolescents are somewhat more likely to become physically active adults [12]. To be able to increase PA levels, it is important to develop interventions that target the most important determinants of PA.

Although socio-ecological models of health behavior have suggested that the physical environment (such as the availability and accessibility of PA opportunities such as parks, sport facilities, bicycle lanes and sidewalks) may be a potent determining factor for PA [13-15], recent reviews of the literature among adolescents [16] as well as adults [17] show that the evidence is not consistent. This indicates that more research is warranted, and that special attention should be directed at studying these associations among adolescents, since their patterns of activity and use of facilities differ from those of adults. Moreover, different conceptualisations of the environment may apply to different population groups[18]. Therefore care should be taken in translating finding from adult literature to adolescents.

An important issue related to the study of environmental influences on PA behavior is the measurement of the physical environment, such as availability and accessibility of physical activity opportunities and barriers in the relevant environment. In recent years more detailed objective measures of PA opportunities have become available, for example, those documented in geographic information systems (GIS). Objective measures are generally regarded as being superior to subjective self-reports. However, adolescents may perceive their environments differently even if they live in the same "objective" environment. For example, a person who is motivated to be physically active may be more likely to perceive more opportunities to be physically active than someone who is less motivated. Also, adolescents who are physically active may be more knowledgeable about the available opportunities than an inactive person.

Earlier research has indicated that there are indeed differences between perceived and objectively measured environments, and both may have different associations with health behaviors. Previous studies have found associations between objective availability of parks [16], recreational facilities[17], commercial PA-related facilities [19] with moderate-to-vigorous PA (MVPA) among adolescents. No associations were found between objective measures of accessibility and walkability and MVPA [20]. Objective measures of walkability [21] and features of school routes [22] were associated with active commuting to school; no associations were found for intersection density [21] and land use [21]. Studies that examined associations between perceived environmental factors and PA behavior have found that perceived availability of recreational facilities [23-25] and perceived access [20] were not associated with MVPA. Perceived availability of walking and bicycle facilities such as sidewalks and bicycle lanes (by parents) aesthetics [21] and street connectivity [21] were, and parental perceived traffic safety was not related to active commuting to school [21,22].

Most of the above-mentioned studies did not explicitly explore differences between objective and perceived environment. A recent paper by Ball et al. [26] found a mismatch between the objective and perceived environment in adults. Another study [25] did compare the perceived environment and the objective environment in an adolescent population and found significant associations between objective and perceived environmental factors. However, for adolescent girls only the perceived environment was related to MVPA. These studies were conducted in an older population [26] or a female-only sample of adolescents [25] and not a general sample of adolescents in Europe. Better insight into these differences between the perceived and objective environment and their associations among adolescents could have important consequences for intervention development, because changing people's perceptions requires other strategies than modifying the actual environment. Perceptions of the environment may be changed by health education techniques by making adolescents aware of possibilities to be active, whereas modifying the actual environment may involve building new parks or sports facilities or enhancing accessibility.

In studying how physical environmental factors influence PA, it is important to study PA sub-domains instead of total PA, since environmental factors may be specific to particular sub-behaviors [18]. Hence, the availability of sports facilities may be important for engaging in sports, but not important for engaging in active transportation. The present study focuses on engagement in sports activities and walking and cycling during leisure time, as these are both important contributors to adolescent PA [27]. The environmental factors examined in relation to these PA sub-domains were objectively measured and perceived availability of facilities for being physically active - such as parks, sports facilities, bicycle lanes and sidewalks - in the neighborhoods where adolescents live.

The aims of the present study are to 1) examine associations of objective and perceived availability of PA facilities with engagement in two leisure time activities: sports and walking and cycling during leisure time, and 2) to explore the degree of agreement and associations between objective and perceived availability of PA facilities.

## Methods

This study used cross-sectional data on PA from a larger study on ENvironmental Determinants of Obesity in Rotterdam SchoolchildrEn (ENDORSE). Rotterdam is the second largest city of the Netherlands, with approximately 600.000 inhabitants of which 46% are of non-Dutch origin [28]. A detailed description of the study protocol is published elsewhere [29]. The ENDORSE study aims to investigate psychosocial and environmental determinants of overweight- and obesity-related behaviors among adolescents from 12 to 15 years of age. The data were collected in 2005-2006. The medical ethics committee of the Erasmus University Medical Center in Rotterdam issued a "declaration of no objection" for the study.

### Sampling and procedure

Schools participating in a health surveillance system (n = 56) conducted by the Rotterdam Public Health Service were invited to take part in the ENDORSE study. Of the 56 schools, 24 schools were willing to participate. These 24 schools were stratified according to the area of the city in which they were located (north, south, east or city centre), to ensure a range of physical and cultural environments. Of the 24 schools willing to participate, 17 were randomly selected for participation. In each selected school, approximately five classes were randomly selected for the study. All adolescents in a class participated, unless they or their parents indicated they were unwilling to do so. A total of 1668 adolescents were invited to take part in the ENDORSE study. In the present study, only the adolescents who lived in neighborhoods of the city of Rotterdam which were not adjacent to other municipalities were eligible for analyses, because objective environmental data was available for them.

A total number of 654 adolescents were included in the present study. Data of the other adolescents who were initially invited to participate were not available due to various reasons. The ENDORSE questionnaire was completed by 1361 adolescents (82%) from 71 classes in 16 schools. During questionnaire completion, 187 adolescents were absent. Data from another 120 adolescents from one school were lost due to a printing mistake. A total of 817 adolescents met the criterion of living in neighborhoods not adjacent to other municipalities. Of these 817 adolescents, 654 (80%) had complete questionnaire data. Compared to the sample with complete questionnaire data, adolescents with missing questionnaire data were significantly older. A significantly higher percentage of adolescents with missing data were of non-Western decent and attended the lower school levels.

During one school hour, the adolescents completed a printed questionnaire on dietary and PA behaviors and potential determinants in the presence of a research assistant and a teacher.

### Measures

#### Background characteristics

Date of birth, gender, country of birth (of the adolescent and the father and mother) and zip code of the home address were assessed in the questionnaire. School level was provided by the school and was categorized into senior general secondary education (i.e. preparatory education for university) and vocational education. A variable for ethnicity was calculated from the questions on country of birth according to the Statistics Netherlands standard. An adolescent was considered to be of Western descent if he or she and both parents were born in the Netherlands, another European country, Oceania, North America, Indonesia or Japan. If the adolescent or one of the parents was born in another country, the adolescent was considered to be of non-Western descent. Exact age was calculated by subtracting the reported date of birth from the date of measurements.

#### Physical activity

PA was assessed by means of an adapted version of the Activity QUestionnaire for Adolescents & Adults (AQUAA) (Chin A Paw MJ, et al, paper under review). The AQUAA is a 7-day recall questionnaire that consists of items on frequency and time engaged in PA at school and during leisure time, active transport to school and during leisure time and sedentary behaviors during leisure time. This questionnaire showed fair to moderate test-retest reproducibility, with intra-class correlations ranging from 0.46 to 0.59.

The present study used the questionnaire items for assessing sports and walking and cycling during leisure time. Engagement in sports was assessed by asking adolescents to write down up to three sports activities in which they participated regularly and to indicate on how many days of the week they engaged in this activity. An overall measure for frequency of engagement in sports activities was created by summing up the number of days reported for the three sports activities. This variable was dichotomized into a variable for "engaging in sports at least three times a week" yes (1) or no (0). The cutoff point used is in agreement with the criteria for complying with the fitness norm (engaging in sports activities at least three times a week [3,4]). Leisure-time walking and cycling were assessed by two items that determine the frequency in days and average time spent on the activity per occasion (e.g. "How many days a week do you walk during leisure time?" and "On a day that you walk, how long do you walk on average during leisure time?"). For both variables, the average minutes per day spent on doing these activities was calculated using the following formula:



A composite variable for walking and cycling in leisure time was calculated by adding up the average time spent walking and cycling. This variable was dichotomized, using a cutoff value of 30 minutes a day. This cut-off seems sensible, since spending 30 minutes or more a day on walking and cycling during leisure time constitutes 50% of the recommended level of at least 60 minutes of PA a day.

#### Perceived physical environment

Perceived and objective availability of PA facilities were assessed for the neighborhood in which an adolescent lived. Availability of sidewalks and bicycle lanes was assessed with the items "In my neighborhood most of the streets have a sidewalk" and "There are a lot of bicycle lanes in my neighborhood" with a 5-point scale answering format (completely agree - completely disagree). Because of skewness, these two variables were dichotomized with the median as the cut-off value. The availability of parks and sports facilities was measured using a yes/no answering format, with the questions "Is there a park in your neighborhood?" and "Are there sports facilities in your neighborhood?". We chose to assess facilities in the home neighborhood, since it is likely that adolescents spend a significant part of their leisure time close to their homes [30].

#### Objective physical environment

Objective data on the availability of environmental opportunities to be active in the neighborhood in which the adolescents lived was retrieved from two separate databases, both managed by the municipality of Rotterdam. The objective availability of PA facilities was retrieved from a GIS database. This database contains the geographical coordinates of parks and public sports facilities (including sports halls, skate parks, fitness centers and swimming pools). Addresses of participants were "geocoded" using the centroid of their 6-digit zip codes. Crow-fly distances were used to assess the number of facilities within a 1500-meter radius of the centroid of the 6-digit zip codes, based on recommendations by Colabianchi et al[31] The number of parks and sports facilities within this radius was counted using ArcGIS 9.3 to form separate continuous variables. Availability of parks was defined as having a border of the park within a 1500 meter radius of the adolescents' home address. In addition to the continuous variables, new dichotomous variables for availability (0 = not present - 1 = present) were subsequently calculated from these counts.

Information for calculating an objective measure for availability of sidewalks and bicycle lanes was retrieved from another municipal database containing information on the total area of sidewalks and bicycle lanes per zip code defined neighborhood as well as the total land area per zip code defined neighborhood. The percentage of the area of sidewalks and bicycle lanes of the total land area in a neighborhood was calculated for the zip code defined neighborhoods. These variables were linked to the adolescents' home address zip code. In addition to these continuous variables, new dichotomized variables were created with the median as cut-off value. Complete environmental data was available for adolescents for which the 1500-meter radius was within the municipality borders of Rotterdam.

### Analyses

Descriptive statistics were used to describe the study population. Multi-level multivariate logistic regression analyses (MLwiN 2.02) were used to examine the associations between objective and perceived environment and PA. A two-level structure was used, with census defined neighborhood and adolescent as the levels. The census defined neighborhood was chosen as a level to account for clustering within the neighborhoods. Objective and perceived environmental factors were entered in separate regression analyses. Engaging in sports more than three times a week was regressed on objective and perceived measures of availability of sports facilities, parks, sidewalks and bicycle lanes. Engaging at least 30 minutes a day in walking and cycling during leisure time was regressed on objective and perceived measures of the availability of parks, sidewalks and bicycle lanes in two separate models. All models were adjusted for age, gender, ethnicity and educational level.

Cohen's kappa and percentage agreement between objective and perceived availability of PA facilities were calculated using the dichotomized environmental variables, to explore the level of agreement between these variables. Kappa values higher than 0.40 were considered to reflect a fair agreement[32] Percentage agreement higher than 75% was considered to reflect a good agreement. Associations of the continuous objective environmental factors with perceived environmental factors were assessed by univariate logistic regression analyses, with perceived environment as dependent variable. The above-mentioned analyses were conducted in SPSS 11.

For all tests, a result was considered significant if the p-value was lower than 0.05 for a two-sided test.

## Results

### Participants

The mean age of the participants in this study was 14.1 (+/- 1.2), 48.9% was male, 53.8 attended vocational education (Table 1). See Table 1 for more background data.

**Table 1:** Description of the final sample

N	654
Male (%)	48.9
Average age (SD)	14.1 (+/- 1.2)
Western ethnic background (%)	41.1
Educational level	
Vocational education (%)	53.8
Senior general secondary education (%)	46.2
Engaging in sports at least three times a week (%)	62.2
Engaging in walking and cycling during leisure time at least 30 minutes a day (%)	80.3
	
*Perceived environment*	
Parks available (% yes)	73.5
Sports facilities available (% yes)	73.4
Sidewalks available (% a lot)	94.3
Bicycle lanes available (% a lot)	35.5
	
*Objective environment*	
Availability of parks^a ^(SD)	0.95 (+/- 0.8)
Availability of sports facilities^a ^(SD)	14.7 (+/- 7.5)
Availability of sidewalks^b ^(SD)	13.2 (+/- 5.8)
Availability of bicycle lanes^b ^(SD)	1.1 (+/- 0.5)

^a ^Number within a radius of 1500 meters of a participants' home; ^b ^percentage of neighborhood land area

#### Associations of environmental factors with sports and with walking and cycling in leisure time

Multivariate analyses (Table 2) show that adolescents who perceived that sports facilities were available in their neighborhood had higher odds to engage in sports activities more than three times a week (OR:1.7, 95% CI: 1.2-2.6). Table 3 shows that adolescents who reported that there were parks in their neighborhood had higher odds to walk and/or cycle at least 30 minutes a day in leisure time (OR: 1.7, 95% CI: 1.1-2.6). No associations were found between objective measures of the environment and sports or walking and cycling during leisure time.

**Table 2:** Odds ratios (OR) and 95% confidence intervals (CI) for engaging in sports activities at least three times a week

	**Model 1: Demographics**	**Model 2: Perceived environment** **(N = 654)**	**Model 3: Objective environment** **(N = 654)**
	**OR [95% CI]**	**OR [95% CI]**	**OR [95% CI]**
Gender (referent = male)	**0.30 [0.21-0.42]**	**0.27 [0.19-0.38]**	**0.28 [0.20-0.40]**
Age	**0.81 [0.71-0.94]**	**0.78 [0.68-0.91]**	**0.82 [0.70-0.95]**
Ethnicity (referent = Western)	1.01 [0.72-1.42]	1.08 [0.76-1.53]	1.13 [0.76-1.67]
Educational level (referent = high)	1.06 [0.76-1.48]	1.08 [0.77-1.53]	1.04 [0.73-1.48]
			
*Environment*			
Parks^a, b^		1.13 [0.76-1.67]	1.06 [0.83-1.35]
Sports facilities^a, b^		**1.73 [1.16-2.56]**	0.98 [0.96-1.01]
Bicycle lanes^a, c^		1.08 [0.76-1.54]	1.15 [0.78-1.70]
Sidewalks^a, c^		1.08 [0.53-2.21]	0.99 [0.96-1.03]

^a ^For perceived environment, reference is "not available;" ^b ^for objective environment, the number counted within a radius of 1500 meters; ^c ^for objective environment, the percentage of neighborhood land area. Models 2 and 3 are independent of each other. Bold values are significant.

**Table 3:** Odds ratios (OR) and 95% confidence intervals (CI) for engaging in walking and cycling during leisure time at least 30 minutes a day

	**Model 1: Demographics**	**Model 2: Perceived environment** **(N = 654)**	**Model 3: Objective environment** **(N = 654)**
	**OR [95% CI]**	**OR [95% CI]**	**OR [95% CI]**
Gender (referent = male)	**0.53 [0.35-0.80]**	**0.51 **[0.34-0.76]	**0.53 [0.36-0.80]**
Age	1.01 [0.86-1.19]	1.01 [0.85-1.20]	1.02 [0.87-1.21]
Ethnicity (referent = Western)	**1.98 [1.32-2.97]**	**2.03 [1.36-3.04]**	**1.75 [1.13-2.72]**
Educational level (referent = high)	1.32 [0.89-1.97]	1.35 [0.91-2.01]	1.30 [0.88-1.95]
			
*Environment*			
Parks^a, b^		**1.66 [1.07-2.57]**	0.97 [0.74-1.27]
Bicycle lanes^a, c^		1.16 [0.76-1.77]	1.01 [0.64-1.60]
Sidewalks^a, c^		1.58 [0.73-3.40]	1.03 [0.99-1.07]

^a ^For perceived environment, reference is "not available;" ^b ^for objective environment, the number counted within a radius of 1500 meters; ^c ^for objective environment, the percentage of neighborhood land area. Models 2 and 3 are independent of each other. Bold values are significant.

#### Agreement between objectively measured and perceived environmental factors

Kappa values for agreement between objective and perceived availability of parks, sports facilities, bicycle lanes and sidewalks were low (0.00 - 0.08) (Table 4). The % agreement between objective and perceived environment was low to moderate, ranging from 53.8% for sidewalks to 73.0% for sports facilities. Univariate regression analyses showed that the odds of perceiving parks to be available was higher when more parks were present (OR: 1.5, 95% CI: 1.2-1.9) (Table 4).

**Table 4:** Kappa statistics and % agreement between perceived and objective environmental measures and odds ratios for perceiving availability of facilities

**Facility**	**Kappa**	**% Agreement**	**% Over-reporting**	**% Under-reporting**	**OR [95% CI]**^**c**^
Parks^a^	0.045	62.3	19.6	18.2	**1.51 [1.18-1.93]**
Sports facilities^a^	-0.004	73.0	0.6	26.5	0.99 [0.97-1.01]
Sidewalks^b^	-0.005	53.8	43.0	3.2	1.02 [0.97-1.08]
Bicycle lanes^b^	0.053	54.1	18.3	27.5	1.40 [0.98-1.99]

^a ^For objective environment, the number counted within a radius of 1500 meters; ^b ^for objective environment, the percentage of neighborhood land area. Bold values are significant; ^c ^associations perceived environment with objective environment

## Discussion

This study explored associations between adolescent perceptions and objectively assessed availability of PA facilities in the neighborhood in which they lived with sports and walking and cycling in leisure time among an adolescent sample in the Netherlands. The results show that adolescents who perceived higher availability of sports facilities in their home neighborhood were more likely to report engaging in sports at least three times a week. Adolescents who perceived a higher availability of parks in their neighborhood were more likely to engage in walking and cycling during leisure time for at least 30 minutes a day. No associations were found between objectively assessed availability of sports facilities and parks and PA. This study also explored the degree of agreement between "perceived" and "objective" availability of PA facilities in adolescents' home neighborhoods. Agreement between objective and perceived availability of facilities was low. It may be that both measures of the environment are truly different constructs, but it may also be partly attributed to the measurement of both constructs.

The present study indicates that perceptions of PA facilities may be more strongly related to PA than objective measures of such opportunities. This finding is consistent with the results of Scott et al[25]. In a sample of adolescent girls, they found that perceived availability of recreational facilities was related to MVPA but objective availability was not. Maddison et al. found comparable results in that perceived environmental measures were related to self-reported MVPA, but objective measures were not related to MVPA in adolescents [20]. Based on socio-ecological models such as the EnRG framework, also a direct relation between the objective 'real' environment and behavior would be expected, through a more or less 'mindless' or automatic response triggered by environmental cues and opportunities [13,33,34]. However, the findings of this study and previous studies do not indicate that such a mindless or automatic response is triggered by the PA facilities in the home neighborhood that we included in our study. Nevertheless, the objective environment is expected to play a role, since perceptions of availability are likely to be the result of an interpretation and cognitive processing of what is actually out there. Therefore, it may be that the objective environment facilitates behavior but is not sufficient to let people actually perform the behavior [35]. Another explanation for finding an association between perceived availability and not objective availability and behavior is that perception of the environment is in the cognitive domain, just like other cognitions such as attitudes and intention that may be associated with behavior. Perception of facilities could then be considered as a more proximal correlate of behavior than the objective environment. Another important issue to note is the low agreement between perceptions and objective measures of the PA facilities. This low agreement may indicate that there is a mismatch between objective and perceived availability of facilities. This may also partly explain why we found an association of the aspects of the perceived environment with behavior, while we did not find these associations for objective environment and behavior. Even though there are no studies to confirm these findings for adolescents, these results are in line with the findings of studies conducted among adults [26,36-38], and adds to the notion of McGinn et al[36] that objective and perceived environmental factors are different constructs. It may therefore be that people living in the same objective environment have different perceptions of the same environment. Indeed, studies in adult populations showed that perceptions of the environment may depend on individual and environmental characteristics [18] e.g. access to vehicles and public transportation [39] and peoples' willingness to travel.

It is important that future studies examine in more detail which factors influence perceptions of the physical environment among adolescents and which factors may potentially moderate or confound the associations between environment and behavior. For adolescents, other aspects of the environment, such as the social or cultural environment (i.e., what their friends do, what is accepted), the information environment, the organizational environment (what sports activities are organized for adolescents in the available sports facilities) and their interactions [40] may play a role in forming perceptions and influencing PA behavior. Another issue of relevance that needs to be addressed in future research among adolescents is the potential interaction between availability of facilities (objective and perceived) and motivation and the influence on PA behavior. For instance, an interaction with motivation can be expected as has been observed with the perceived environment × intention interaction in adults [41]. There may also be interaction between perceived environment and other cognitions, such as for example demonstrated in a study by Haug and colleagues [42] that showed interactions between cognitions (i.e. adolsescents' interests in participating in physical activity) and perceived availability of facilities. If future research further confirms that perceptions are more important in directly predicting adolescent PA behavior than the actual environment, interventions to promote adolescent PA should take this into account.

This study also provides some evidence for the postulation that the environment has behavior-specific associations with PA [18] and that it is important to study environmental factors related to specific PA sub-domains. In this study, we found that different perceived environmental factors were associated with sports than with walking and cycling during leisure time. The importance of studying relevant environmental factors for specific PA sub-domains has previously been suggested for studies among adults [43,44]. It may even be that the relevant distance to certain destinations is behavior-specific, as was found among adults [45]. Future studies should examine if this is also the case among adolescents.

This study has some limitations. One important limitation is the cross-sectional design. It is thus not possible to draw causal inferences. Therefore, it may be the case that adolescents who engage more in sports or walking and cycling perceive more PA facilities because they use them more often. Longitudinal studies and (natural) experiments are needed to gain a better understanding of prediction and causal pathways. Cross-sectional studies are an efficient manner to explore issues to help to define hypotheses for further studies using stronger research designs, and that is what this study aimed for. Another limitation is the reliance on self-reported measures of PA, which may have introduced bias. The analyses were conducted in an urban sample of adolescents. Of them, adolescents of non-Western decent, lower educational levels and those who were older were more likely to have missing data. Therefore, care should be taken in translating these results to other populations. The explorative analysis, comparing the objective and perceived environment, has some limitations as well. The availability of parks and sports facilities was analyzed using crow-fly distances and the availability of bicycle lanes and sidewalks was analyzed using zip code defined neighborhoods. These measures may not fully match the perceived environment of adolescents in this study [46]. Differences between the perceived and objective environment found in this study may be due to this possible discrepancy.

To conclude, we found that the perceived availability of parks was associated with leisure time walking and cycling and the perceived availability of sports facilities was associated with engaging in sports. The objectively assessed availability did not show associations with walking and cycling in leisure time or self-report frequency of sports participation. Modifying the perception of the availability of parks and sports facilities may be a useful strategy in interventions aimed at improving PA among adolescents. This study also suggests that the objective and perceived physical environment are different constructs. Future research should use better conceptualizations of the perceived and objective neighborhood to confirm these explorative findings.

## Competing interests

The authors declare that they have no competing interests.

## Authors' contributions

RGP carried out the study and conducted the data-analysis and drafted the manuscript.

AO and KvdH designed and conducted the ENDORSE study, participated in discussing the paper, provided methodological input, and helped to draft the manuscript. JB designed the study and helped to draft the manuscript. All authors read and approved the final manuscript.

## References

[B1] Physical activity fundamental to preventing diseases. U.S. Department of Health and Human Services, Office of the Assistant Secretary for Planning and Evaluation, 2002. http://aspe.hhs.gov/health/reports/physicalactivity/physicalactivity.pdf.

[B2] the European Heart Initiative (2001). Children and Young People - the Importance of Physical Activity.

[B3] Kemper HCG, Ooijendijk WTM, Hildebrandt VH, Ooijendijk WTM, Stiggelbout M, Hopman-Rock M (2004). De Nederlandse Norm voor Gezond Bewegen. Trendrapport Bewegen en Gezondheid 2002/2003.

[B4] (1998). American College of Sports Medicine Position Stand. The recommended quantity and quality of exercise for developing and maintaining cardiorespiratory and muscular fitness, and flexibility in healthy adults. Med Sci Sports Exerc.

[B5] Roberts C, Tynjälä J, Komkov A, Currie C, Roberts C, Morgan A, Smith R, Settertobulte W, Samdal O, Barnekow Rasmussen V (2004). Physical Activity. Young people's health in context Health Behaviour in School-aged Children (HBSC) study: international report from the 2001/2002 survey.

[B6] StatLine. http://statline.cbs.nl/StatWeb/.

[B7] Scully M, Dixon H, White V, Beckmann K (2007). Dietary, physical activity and sedentary behaviour among Australian secondary students in 2005. Health Promot Int.

[B8] Prevention CfDCa (2006). Youth Risk Behavior Surveillance - United States, 2005. MMWR Surveill Summ.

[B9] Tammelin T, Ekelund U, Remes J, Nayha S (2007). Physical activity and sedentary behaviors among Finnish youth. Med Sci Sports Exerc.

[B10] Lampert T, Mensink GB, Romahn N, Woll A (2007). [Physical activity among children and adolescents in Germany. Results of the German Health Interview and Examination Survey for Children and Adolescents (KiGGS)] Korperlich-sportliche Aktivitat von Kindern und Jugendlichen in Deutschland. Ergebnisse des Kinder- und Jugendgesundheitssurveys (KiGGS). Bundesgesundheitsblatt Gesundheitsforschung Gesundheitsschutz.

[B11] Boreham C, Riddoch C (2001). The physical activity, fitness and health of children. J Sports Sci.

[B12] Telama R, Yang X, Viikari J, Valimaki I, Wanne O, Raitakari O (2005). Physical activity from childhood to adulthood: a 21-year tracking study. Am J Prev Med.

[B13] Kremers SP, de Bruijn GJ, Visscher TL, van Mechelen W, de Vries NK, Brug J (2006). Environmental influences on energy balance-related behaviors: A dual-process view. Int J Behav Nutr Phys Act.

[B14] Elder JP, Lytle LA, Sallis JF, Rohm Young D, Steckler A, Simons-Morton D, Stone EJ, Jobe JB, Stevens J, Lohman T (2006). A description of the social-ecological framework used in the trial of activity for adolescent girls (TAAG). Health Education Research.

[B15] Panter JR, Jones AP, van Sluijs EM (2008). Environmental determinants of active travel in youth: A review and framework for future research. Int J Behav Nutr Phys Act.

[B16] Cohen DA, Ashwood JS, Scott MM, Overton A, Evenson KR, Staten LK, Porter D, McKenzie TL, Catellier D (2006). Public parks and physical activity among adolescent girls. Pediatrics.

[B17] Gordon-Larsen P, Nelson MC, Page P, Popkin BM (2006). Inequality in the built environment underlies key health disparities in physical activity and obesity. Pediatrics.

[B18] Giles-Corti B, Timperio AF, Bull FC, Pikora T (2005). Understanding physical activity environmental correlates: increased specificity for ecological models. Exercise and sport sciences reviews.

[B19] Powell LM, Chaloupka FJ, Slater SJ, Johnston LD, O'Malley PM (2007). The availability of local-area commercial physical activity-related facilities and physical activity among adolescents. Am J Prev Med.

[B20] Maddison R, Hoorn S Vander, Jiang Y, Ni Mhurchu C, Exeter D, Dorey E, Bullen C, Utter J, Schaaf D, Turley M (2009). The environment and physical activity: the influence of psychosocial, perceived, and built environmental factors. Int J Behav Nutr Phys Act.

[B21] Kerr J, Rosenberg D, Sallis JF, Saelens BE, Frank LD, Conway TL (2006). Active commuting to school: Associations with environment and parental concerns. Med Sci Sports Exerc.

[B22] Timperio A, Ball K, Salmon J, Roberts R, Giles-Corti B, Simmons D, Baur LA, Crawford D (2006). Personal, family, social, and environmental correlates of active commuting to school. Am J Prev Med.

[B23] Evenson KR, Scott MM, Cohen DA, Voorhees CC (2007). Girls' perception of neighborhood factors on physical activity, sedentary behavior, and BMI. Obesity.

[B24] Mota J, Almeida M, Santos P, Ribeiro JC (2005). Perceived Neighborhood Environments and physical activity in adolescents. Prev Med.

[B25] Scott MM, Evenson KR, Cohen DA, Cox CE (2007). Comparing perceived and objectively measured access to recreational facilities as predictors of physical activity in adolescent girls. Journal of Urban Health.

[B26] Ball K, Jeffery RW, Crawford DA, Roberts RJ, Salmon J, Timperio AF (2008). Mismatch between perceived and objective measures of physical activity environments. Prev Med.

[B27] de Vries C, Bik M (2006). Quickscan Rotterdamse jongeren in hun vrije tijd.

[B28] Key figures Rotterdam 2006. http://cos.rotterdam.nl/Rotterdam/Openbaar/Diensten/COS/Publicaties/PDF/KC2006UK.pdf.

[B29] Horst K van der, Oenema A, Looij-Jansen P van de, Brug J (2008). The ENDORSE study: research into environmental determinants of obesity related behaviors in Rotterdam schoolchildren. BMC Public Health.

[B30] Ries AV, Gittelsohn J, Voorhees CC, Roche KM, Clifton KJ, Astone NM (2008). The environment and urban adolescents' use of recreational facilities for physical activity: a qualitative study. Am J Health Promot.

[B31] Colabianchi N, Dowda M, Pfeiffer KA, Porter DE, Almeida MJ, Pate RR (2007). Towards an understanding of salient neighborhood boundaries: adolescent reports of an easy walking distance and convenient driving distance. Int J Behav Nutr Phys Act.

[B32] Landis J, Koch G (1977). The measurement of observer agreement for categorical data. Biometrics.

[B33] Aarts H, Dijksterhuis A (2000). The automatic activation of goal-directed behaviour: the case of travel habit. Journal of Environmental Psychology.

[B34] Aarts H, Paulussen T, Schaalma H (1997). Physical exercise habit: on the conceptualization and formation of habitual health behaviours. Health Education Research.

[B35] Giles-Corti B, Donovan RJ (2003). Relative influences of individual, social environmental, and physical environmental correlates of walking. Am J Public Health.

[B36] McGinn AP, Evenson KR, Herring AH, Huston SL, Rodriguez DA (2007). Exploring associations between physical activity and perceived and objective measures of the built environment. J Urban Health.

[B37] Kirtland KA, Porter DE, Addy CL, Neet MJ, Williams JE, Sharpe PA, Neff LJ, Kimsey CD, Ainsworth BE (2003). Environmental measures of physical activity supports. Perception versus reality. Am J Prev Med.

[B38] Boehmer TK, Hoehner CM, Deshpande AD, Brennan Ramirez LK, Brownson RC (2007). Perceived and observed neighborhood indicators of obesity among urban adults. Int J Obes (Lond).

[B39] Ball K, Timperio AF, Crawford DA (2006). Understanding environmental influences on nutrition and physical activity behaviors: where should we look and what should we count?. Int J Behav Nutr Phys Act.

[B40] Sallis JF, Cervero RB, Ascher W, Henderson KA, Kraft MK, Kerr J (2006). An ecological approach to creating active living communities. Annual review of public health.

[B41] Rhodes RE, Courneya KS, Blanchard CM, Plotnikoff RC (2007). Prediction of leisure-time walking: an integration of social cognitive, perceived environmental, and personality factors. Int J Behav Nutr Phys Act.

[B42] Haug E, Torsheim T, Samdal O (2008). Physical environmental characteristics and individual interests as correlates of physical activity in Norwegian secondary schools: The health behaviour in school-aged children study. Int J Behav Nutr Phys Act.

[B43] De Bourdeaudhuij I, Sallis JF, Saelens BE (2003). Environmental correlates of physical activity in a sample of Belgian adults. Am J Health Promot.

[B44] King AC, Toobert D, Ahn D, Resnicow K, Coday M, Riebe D, Garber CE, Hurtz S, Morton J, Sallis JF (2006). Perceived environments as physical activity correlates and moderators of intervention in five studies. Am J Health Promot.

[B45] McCormack GR, Giles-Corti B, Bulsara M (2008). The relationship between destination proximity, destination mix and physical activity behaviors. Prev Med.

[B46] Coulton CJ, Korbin J, Chan T, M S (2001). Mapping residents' perceptions of neighborhood boundaries: a methodological note. American Journal of Community Psychology.

